# How fast is a collective bacterial state established?

**DOI:** 10.1371/journal.pone.0180199

**Published:** 2017-06-23

**Authors:** Mikkel Lindstrøm Sørensen, Peter Dahl, Thomas Sams

**Affiliations:** 1 Biomedical Engineering, Dept. of Electrical Engineering, Technical University of Denmark, DK-2800 Lyngby, Denmark; 2 Dept. of Applied Mathematics and Computer Science, Technical University of Denmark, DK-2800 Lyngby, Denmark; Niels Bohr Institute, DENMARK

## Abstract

Bacteria in a biofilm colony have the capacity to monitor the size and growth conditions for the colony and modify their phenotypical behaviour to optimise attacks, defence, migration, etc. The quorum sensing systems controlling this involve production and sensing of diffusive signal molecules. Frequently, quorum sensing systems carry a positive feedback loop which produces a switch at a threshold size of the colony. This all-or-none switch can be beneficial to create a sudden attack, leaving a host little time to establish a defence. The reaction-diffusion system describing a basal quorum sensing loop involves production of signal molecules, diffusion of signal molecules, and detection of signal molecules. We study the ignition process in a numerical solution for a basal quorum sensor and demonstrate that even in a large colony the ignition travels through the whole colony in a less than a minute. The ignition of the positive feedback loop was examined in different approximations. As expected, in the exact calculation the ignition was found to be delayed compared to a calculation where the binding of signal molecules was quasistatic. The buffering of signal molecules is found to have little effect on the ignition process. Contrary to expectation, we find that the ignition does not start when the threshold is reached at the center—instead it allows for the threshold to be approached in the whole colony followed by an almost simultaneous ignition of the whole biofilm aggregate.

## Introduction

Quorum sensing (QS) is a biological regulation process utilised by bacteria to control behaviour in accordance with size, density, and growth-rate of a bacterial population [1]. The process is based on diffusible signal molecules, produced by the bacteria at a background level. The signal molecules are able to bind to regulator molecules within the bacteria, thereby activating the regulator [2].

The collective behaviour regulated by QS was reported in *Vibrio fischeri* where it regulates camouflage light in large cell colonies in the host [3–5]. Since then, QS systems have been reported in many bacteria, e. g. *Aeromonas hydrophila* [6–9], *Agrobacterium tumefaciens* [10], and *Pseudomonas aeruginosa* [11–13]. The presence of QS in colonies of bacteria appears to be the rule rather than the exception.

Frequently, the array of gene expressions acting under the control of activated QS regulators includes signal molecule synthetase. This positive feedback leads to a size-sensitive switch which can be used to control collective behaviour [1–5, 14]. The switch makes it possible to maintain an invisible state until the sudden QS regulated attack sets in. Recently, a proper measure of the “size” of a spherical biofilm aggregate was established as the cell density multiplied by the squared radius of the colony [15, 16]. The establishment of the size measure was based on the observation that the concentration of the activated regulator

$$r_{a}=\left[R_{2}S_{2}\right]$$ (1)

may be interpreted as the intrinsic measure of how quorate the state of the colony is and controls the quorum sensing feedback as well as QS regulated genes [15].

In small colonies the signal molecules are produced at a low background level. In larger colonies, the diffusive signal molecules accumulate, activate the transcriptional regulator, and induce transcription of the signal molecule synthetase at an increased level.

The dimer form of the activated regulator implied in Eq (1) allows for a fully developed switch in the ignition of the quorum as observed in Gram negative bacteria [3–5, 14]. The dimeric form is typical to quorum sensors and has been confirmed in main QS loops in a range of Gram negative bacteria [1, 12, 17–25]. In the present study we will study the time course of the ignition of the size-dependent switch in the most basic form of a single quorum sensitive switch.

## Reaction-diffusion system

**Fig 1:**
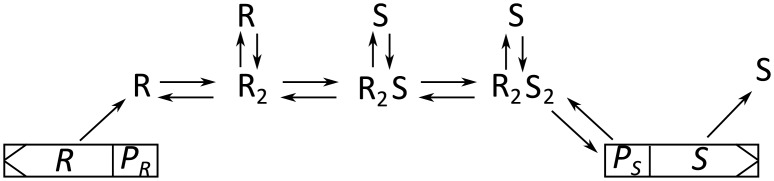
Reaction scheme of a generic quorum sensing process with regulator (R with promoter *P*_*R*_) and signal molecules (S with promoter *P*_*S*_). In this example dimerization takes place prior to signal molecule binding. As the signal molecules produced by the cell itself diffuse quickly away, the signal molecules binding to the regulator typically come from other cells. Figure modified form [15]

The quorum sensing system considered in this article, is based on the reaction-diffusion model proposed by Ferkinghoff-Borg and coworkers [15, 16, 25]. This model examines a dimer based regulator system with concentrations dependent on both spatial coordinates and time in a spherically symmetric geometry. These reaction-diffusion equations are obtained by considering the reactions that occur, when the unactivated regulator molecules dimerize and are subsequently activated by ligand binding as illustrated in Fig 1.

The reactions in Fig 1 are modelled by the four differential Eqs (2)–(5) [15].

$$\frac{\partial r_{1}}{\partial t}=b_{1}+2k_{2}^{-}r_{2}-2k_{2}^{+}r_{1}^{2}-\lambda_{1}r_{1}$$ (2)

$$\frac{\partial r_{2}}{\partial t}=k_{2}^{+}r_{1}^{2}+k_{3}^{-}r_{3}-2k_{3}^{+}r_{2}s-\left(k_{2}^{-}+\lambda_{2}\right)r_{2}$$ (3)

$$\frac{\partial r_{3}}{\partial t}=2k_{3}^{+}r_{2}s+2k_{4}^{-}r_{4}-k_{4}^{+}r_{3}s-\left(k_{3}^{-}+\lambda_{3}\right)r_{3}$$ (4)

$$\frac{\partial r_{4}}{\partial t}=k_{4}^{+}r_{3}s-\left(2k_{4}^{-}+\lambda_{4}\right)r_{4}$$ (5)

Each of the equations model the change in concentration of each regulator stage, with *r*_1_ as the concentration of R, *r*_2_ the concentration of R_2_ and so forth. The final reaction produces the activated dimer regulator concentration *r*_4_ = *r*_*a*_ = [R_2_S_2_].

The term *b*_1_ signifies the production of monomer regulator with concentration *r*_1_ = [R]. In the model the reaction between regulators is governed by on- and off-rates, denoted by $k_{i}^{+}$ and $k_{i}^{-}$, as well as degradation rates *λ*_*i*_, *i* = 1, …, 4. The dimer degradation rates are assumed to be equal *λ*_*d*_ = *λ*_2_ = *λ*_3_ = *λ*_4_ [16] and significantly lower than the monomer decay rate *λ*_1_. All degradation rates include proteolytic degradation as well as dilution by cell division. The degradation rates are assumed to be considerably slower than the on- and off-rates.

Additionally, an equation governing the production and diffusion of signal molecules is required. The change in signal molecule concentration *s* = [S] is modelled using a diffusion equation containing a production term *ρ*_*v*_*κ*_*s*_:

$$\frac{\partial s}{\partial t}=D\Delta s+\rho_{v}\kappa_{s}$$ (6)

$$\kappa_{s}=\frac{\frac{b_{s}}{k_{s}}K_{s}+r_{a}}{K_{s}+r_{a}}k_{s}\approx \left\{\begin{array}{cc} b_{s} & ,\;r_{a}<\frac{b_{s}}{k_{s}}K_{s}\\ k_{s} & ,\;r_{a}>K_{s} \end{array}\right.$$ (7)

Multiplication of the intracellular production *κ*_*s*_ by the volume fraction occupied by cells, *ρ*_*v*_, ensures correct normalisation of the production term. Note that the production term shifts away from background production, *ρ*_*v*_*b*_*s*_, already when the activated regulator level reaches $\frac{b_{s}}{k_{s}}K_{s}$, which was recognised as the ignition point for the feed-back loop [15].

When solving the model numerically, a spherical geometry with radius R will be assumed. Regulator as well as signal molecule concentrations are thus dependent on time, *t*, and distance from center. Additionally, the boundary of the colony is assumed to be absorbing, s(R,t)=0, corresponding to a rapid exchange of the surroundings.

Eq (6) implicitly assumes that the free signal molecule concentration is much larger than the bound signal molecule concentration. If this is not the case, for a calculation of the time course of the binding, it is necessary to account for the bound signal molecules. This results in the following equation.

$$\frac{\partial s}{\partial t}=D\Delta s+\rho_{v}\kappa_{s}+\rho_{v}\left(2k_{4}^{-}r_{4}+k_{3}^{-}r_{3}-2k_{3}^{+}r_{2}s-k_{4}^{+}r_{3}s\right)$$ (8)

Incorporating this behaviour in the model, introduces a buffering of the signal molecules. In the static limit, the added terms vanish and therefore do not alter the results by Ferkinghoff-Borg *et al*. Below, we shall refer to the solution of Eqs (2)–(5) with Eq (8) as the “exact” model, and the solution with Eq (6) as the solution “without buffering”.

In Eq (6) we only consider the generic case, where there is equilibrium between the concentration of signal molecules inside and outside the cells. This is a fair approximation for QS systems mediated by smaller AHL molecules. However, for larger AHL molecules, active efflux pumps have been reported to assist in transporting the autoinducers out of the cell thereby speeding up the ignition process [26, 27]. Modifications representing the active transport across the membrane may therefore have to be introduced to produce a realistic profile of the ignition process in these cases [28].

Quasi-static and static approximations to the solution of the reaction-diffusion system will serve as references for the obtained results. The quasi-static approximation assumes an instant equilibrium between regulator stages Eqs (2)–(5). This leads to a simple relation between signal molecule concentration and activated regulator concentration

$$r_{4}=r_{a}=\frac{s^{2}}{{\tilde{K}}^{2}+s^{2}}\;r_{m}$$ (9)

where *r*_*m*_ is the maximal concentration of the active form of the regulator and $\tilde{K}$ is an effective dissociation constant which sits at the crossing between the asymptotes for large and small signal molecule concentrations [29]. Both *r*_*m*_ and $\tilde{K}$ are determined by the static solution of Eqs (2)–(5). The quasi-static approximation does not assume the diffusion equation to be in static equilibrium.

The static approximation of the model leads to an instantaneous switch and can be solved by letting all time derivatives vanish [15]. At the center of the colony, the static approximation yields a simple factorised expression for the size of a colony, Σ,

$$\Sigma =\frac{1}{3}R^{2}\rho_{v}=\frac{2D\tilde{K}}{b_{s}}\;\underset{\text{feedback}\;\text{switch}}{\underset{︸}{\frac{b_{s}}{k_{s}}\frac{K_{s}+r_{a}}{\frac{b_{s}}{k_{s}}K_{s}+r_{a}}}}\;\underset{\text{forward}\;\text{switch}}{\underset{︸}{{\left(\frac{r_{a}}{r_{m}-r_{a}}\right)}^{1/2}}}$$ (10)

dependent on the activated regulator concentration, *r*_*a*_, [15]. Here all geometrical properties (density and radius of the colony) are conveniently on the lhs. of the equation, and all intracellular properties are on the rhs. The braces enhance the role of each term in the factorised form. This factorized expression assumes not only time independence but also constant activation throughout the micro colony. Nevertheless, it proves to describe the qualitative features of the system.

The system of differential equations was solved using the numerical solver *pdepe* in Matlab [30, 31]. The initial condition was established by keeping the cell density fixed at a value below the ignition point and allowing the system develop to stationary state. Growth of the colony size, Σ, was accomplished by increasing the cell density, *ρ*_*v*_, at a rate *λ*_0_ = 0.5 h^−1^. The full set of parameters used in the simulation is listed in Table 1.

**Table 1:** Table of parameters.

Parameter	Value	Description	Reference
*b*_1_	1000 nM/h	Monomer regulator production rate	[32, 33]
*D*	∼ 2 mm^2^/h	Diffusion constant	[15, 34]
*b*_*s*_	3600 nM/h	Background production of S	[33]
*k*_*s*_	100 *b*_*s*_	Maximum production of S	[15]
*K*_*s*_	1 nM	Promoter site *P*_*S*_ dissociation constant	Current study
*r*_1_		R concentration	
*r*_2_		R_2_ concentration	
*r*_3_		R_2_S concentration	
*r*_4_ = *r*_*a*_	< *r*_*m*_	R_2_S_2_ activated regulator concentration	
*r*_*m*_	537 nM	Maximal *r*_*a*_ concentration	
$\tilde{K}$	208 nM	Effective dissociation constant	[9, 35–37]
R	100 *μ*m	Radius of colony	[38–41]
*ρ*_*v*_		Cell density (volume fraction occupied by cells)	
$k_{2}^{-}$	1000 h^−1^	2R ← R_2_ rate	[33]
$k_{2}^{+}$	0.5 nM^−1^ h^−1^	2R → R_2_ rate constant	[33, 42, 43]
$k_{3}^{-}$	1000 h^−1^	R_2_ + S ← R_2_S rate	[33]
$k_{3}^{+}$	100 nM^−1^ h^−1^	R_2_ + S → R_2_S rate constant	[33]
$k_{4}^{-}$	1000 h^−1^	R_2_S + S ← R_2_S_2_ rate	[33]
$k_{4}^{+}$	100 nM^−1^ h^−1^	R_2_S + S → R_2_S_2_ rate constant	[33]
*λ*_1_	20 h^−1^	Monomer decay rate	[17, 18]
*λ*_*d*_	0.5 h^−1^	Dimer decay rate (*λ*_2_, *λ*_3_, *λ*_4_)	[1, 24, 25]
*λ*_0_	0.5 h^−1^	cell density growth rate	

Table containing descriptions and values of parameters used in the model.

## Results and analysis

**Fig 2:**
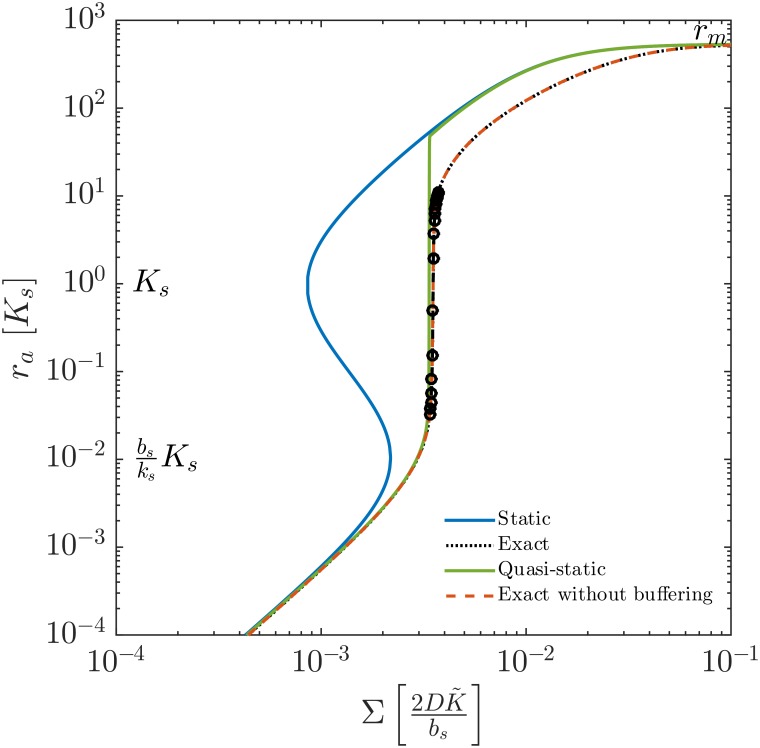
Plot of activated regulator concentration, *r*_*a*_ = *r*_4_, at the center of the colony as a function of geometric size measure, Σ. Both axes are in natural units. The exact solution depicts the solution found when solving Eqs (2)–(5) and (8), whereas non-buffered solution uses Eq (6). The quasi-static solution uses Eqs (6) and (9). The ignition point, $r_{a}=\frac{b_{s}}{k_{s}}K_{s}=10^{-2}K_{s}$, is indicated, as well as the dissociation constant *K*_*s*_ and maximum regulator concentration *r*_*m*_. The exact solution is displayed with circles corresponding to 10 second intervals to indicate the speed of the ignition.

In Fig 2, the activated regulator concentration at the center of the colony, *r*_*a*_, is displayed as a function of the size, Σ. The curves represent the full model with and without buffering (broken lines) compared with the static and quasi-static solutions (full lines).

First, we note that the exact model with buffering is indistinguishable from the model without buffering, thus indicating that buffering of the signal molecules does not play a significant role.

In the factorized static approximation by Ferkinghoff-Borg *et al*. the feed-back loop appears to ignite at a somewhat lower size than the full solution and the quasi-static solutions. This is due to the approximation, that the concentration of activated regulator is taken to be equal to its value at the center throughout the colony. It therefore reflects a limitation of the simple factorized model in Eq (10).

The quasi-static solution resembles the exact solution, differing only slightly around the time of ignition. As expected, the ignition of the system occurs prior to the ignition in the exact solution. In contrast to the quasi-static solution, at ignition, the full solution jumps only part of the way up to the factorized static solution. The reason for this difference is that, in the full model, the dimer concentration needs to build up and does so at the same rate as the dilution from growth and degradation in the cells. The exact and quasi-static solutions show an increase of more than two decades in activated regulator concentration, shortly after reaching the ignition point. Examining the circles, it can be seen that the ignition takes less than a minute. (Here we define “ignition” as a jump of 2 orders of magnitude in concentration of activated regulator, *r*_*a*_.)

**Fig 3:**
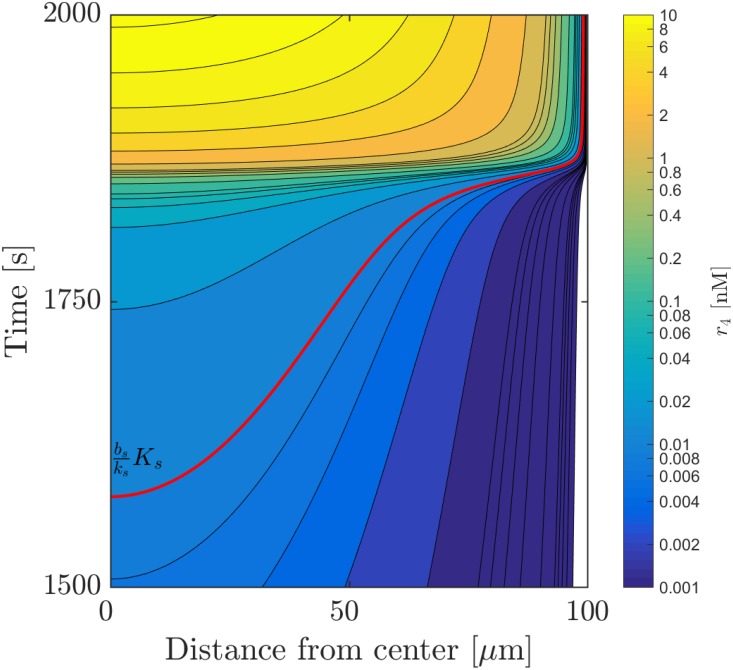
Contour plot of activated regulator concentration *r*_*a*_ = *r*_4_ = [R_2_S_2_] around the time of ignition as a function of time and distance from the center of the colony. The plot contains the exact solution of the system of equations presented in Eqs (2)–(5) as well as Eq (8), i. e. the system describing total signal molecule concentration. The concentration necessary for ignition ($\frac{b_{s}}{k_{s}}K_{s}$ in the static case) is marked by the red contour. A logarithmic colour-scheme has been chosen due to the large range of values pre- and post-ignition.

The activation of the feed-back loop as a function of the radial coordinate and time may be examined in Fig 3. The ignition concentration ($∼\frac{b_{s}}{k_{s}}K_{s}$) is reached at different times throughout the colony, with a difference of approximately 5 minutes between the ignition point at the center and the ignition point close to the boundary. However, the ignition, recognized as dense horizontal lines, does not occur until the ignition condition is reached throughout the whole colony. Once this happens, the whole colony ignites in less than a minute, ignoring a thin region near the boundary which is under the control of the boundary condition. Thus, the contour plot confirms the expectation that the entire colony resides in a well defined state, either on or off.

## Conclusion

We have modelled a generic single-loop quorum sensing system with positive feedback. The primary goal of the study has been to study of the space-time structure of the ignition of the switch produced by the positive feedback in the quorum sensor.

The exact solution exhibits a delayed response compared to both the quasi-static and static approximations, as it is limited by the time it takes the regulator stages to react and build up concentration of activated regulator. Inclusion of buffering terms produces no further retardation of the system.

Using a 3D representation to depict both the spatial and temporal dependency of the activated regulator concentration, the collective behaviour of the colony could be studied. A slow build-up over five minutes to the ignition concentration of activated regulator followed by a quick ignition was observed. The process exhibits the desired behaviour, as the entire colony is either in an on- or off-state. These observations indicate that a partial ignition is difficult to achieve, even for slow systems. The model demonstrates that even the largest naturally occurring biofilm aggregates in chronic infections [41] ignite fully in less than a minute and, truly, can be said to produce a surprise attack.
